# Factors that influence the health status of immigrants living in Greece

**DOI:** 10.3934/publichealth.2020024

**Published:** 2020-05-09

**Authors:** Panayota Sourtzi, Petros Galanis, Olympia Konstantakopoulou, Olga Siskou, Daphne Kaitelidou

**Affiliations:** 1 Sector of Public Health, Department of Nursing, National and Kapodistrian University of Athens, Athens, Greece; 2 Center for Health Services Management and Evaluation, Department of Nursing, National and Kapodistrian University of Athens, Athens, Greece

**Keywords:** immigrants, Greece, self-perceived health, health status, preventive tests

## Abstract

**Aim:**

To examine the health status of immigrants living in Greece and investigate the factors that influence it.

**Methodology:**

A cross-sectional study with 1152 immigrants (response rate = 60%) was conducted during April 2013 to March 2014. Regarding the sampling method, as there is no accurate census of immigrants in Greece the snowball sampling was used. Data collection included demographic characteristics, health status, medication and self-reported preventive health examinations of immigrants (blood count, blood pressure, cholesterol, and blood sugar measurement).

**Results:**

The majority of immigrants originated from Albania (51.4%), while 52.6% were males with mean age 37.6 years. Of those 63.5% were working, 80.9% had legal documents for living and working in Greece and 58.2% had valid health and social security. Most of the immigrants (66.6%) considered their health as good/very good. Immigrants without health insurance, lower monthly family income and worst self-reported health did not adhere with their medication treatment due to cost. Immigrants with legal documents and health insurance performed more often blood count measurement, blood pressure measurement, cholesterol measurement and blood sugar measurement. Increased monthly family income was also associated with higher probability of blood count measurement. Very poor/poor/average self-reported health and increased age were associated with higher probability of taking medicines for chronic diseases.

**Conclusions:**

Self-reported health of immigrants in Greece is good/very good while absence of health insurance and legal documents, lower income and worst self-reported health are associated with worst health outcomes.

## 1. Introduction

Greece has become a host country for a large number of immigrants since the beginning of 90s. The majority of immigrants originate from the Balkans and former Soviet Union countries, but there are also significant numbers of immigrants from south east Asia and Africa [1].

Immigration has become a major issue affecting both the immigrants and the population of host countries. A systematic review found that immigrants suffer more often from mental illnesses in comparison to the native population while lifestyle habits are influenced by the host country population behaviors due to the level of education, the proficiency of the language and the exposure to the local population behaviors [2]. Evidence also shows that immigrants are more vulnerable to social and economic disadvantage, affecting their health status, quality of life, health outcomes and access to health care services [3]–[6]. Thus, immigrants have both, increased health needs and experience significant disparities related to access and use of health services [7],[8].

Primary research regarding immigrants in Greece has focused on the prevalence of health problems carried along from the country of origin and the assessment of health needs in order to organize population specific preventive programs [9]–[11]. Recent studies on immigrants have changed focus and study health behavior [12],[13], health services access and utilization [14]–[17], self-perceived health status [18], and survival and cause-specific mortality [19],[20].

Immigrants in Greece today constitute a considerable part of the population living in the country; their origin is quite diverse and some of the ethnic groups are residents for a considerable duration of time. It is therefore worth studying the way that immigrants perceive their health, the ill-health conditions they face, the preventive measures they follow, the factors that affect their health and the differences within the immigrant population. To the best of our knowledge there is not a single study performed in Greece that address these issues. Therefore, the aim of this study was to fill the gap in this research area, mapping the health status of immigrants living in Greece and investigating the factors that influence it.

## 2. Materials and methods

### 2.1. Study design

A cross-sectional study was conducted and data were collected by means of a questionnaire from April 2013 to March 2014.

### 2.2. Sample

The participants of the study were migrants living in Greece less than 10 years and an effort was made to form a representative group from the migrant population in Greece. According to the 2011 census data immigrants originate from Albania (the vast majority), Pakistan, Bangladesh, Philippines, Afghanistan, Ukraine, Georgia, Nigeria, Ethiopia, Egypt, Moldova (ELSTAT 2011). Immigrants then were grouped for the sake of easier presentation of the results as follows: (a) immigrants from Albania, (b) immigrants from eastern European countries (Ukraine, Moldova, Georgia), (c) immigrants from Asia (Pakistan, Afghanistan, Bangladesh and Philippines and (d) immigrants from Africa (Nigeria, Ethiopia and Egypt).

Snowball sampling was used because there was not possible to draw a random sample from the immigrant population due to the lack of reliable registries for this population. At this stage, oversampling from some ethnic groups was decided in order to obtain more meaningful results. Therefore it was decided to increase the number of immigrants from eastern European countries, Asia and Africa in order to ensure a sufficient sample size from these groups and thus, conduct valid statistical comparisons. Firstly, we approached the key persons in migrant communities in order to facilitate the snowball method and increase the response rate. In fact, these key persons were the leaders or representatives of the immigrants and acted as the contact persons between the researchers and the immigrants. Religious places, markets and workplaces were the main places that interviews with the immigrants took place. Response rate was 60% (1152/1920).

### 2.3. Questionnaire

In order to develop an appropriate tool for data collection an extensive literature review was performed in international (MEDLINE, EMBASE, SCOPUS, CINAHL, DAI) and Greek databases (ΙΑΤRΟΤΕΚ and the National Documentation Centre). The study questionnaire included demographic characteristics, health status, medication and self-reported preventive health examinations of immigrants (blood count, blood pressure, cholesterol, and blood sugar measurement). More information about the development of the questionnaire could be found in the qualitative study and the pilot study previously performed [16],[21].

### 2.4. Process and ethics

The study protocol was approved by the ethics committee of the Faculty of Nursing. University of Athens (Date of approval 03/07/2013, number of approval 115). Participants were informed in detail about the aim and the methodology of the study before completing the questionnaire, which was anonymous and to be completed on a voluntary basis. Completed questionnaires were returned in sealed envelopes to which only the researchers had access.

### 2.5. Statistical analysis

The outcomes of the study were the following: (a) failure to take medication due to cost, (b) self-reported preventive health examinations of immigrants (blood count, blood pressure, cholesterol, and blood sugar measurement) and (c) medication for chronic diseases. The demographic characteristics of the immigrants (e.g. country of origin, gender, age, duration of stay in Greece, valid health insurance, legal documents etc.) and the health status characteristics of immigrants (e.g. self-reported health status, smoking, exercise etc.) constituted the independent variables of the study. Measurement of the independent variables and the outcomes was performed with closed-ended questions through the interviews with the immigrants e.g. “Did you measure your blood pressure the last two years?” with possible answers “Yes, it was abnormal”, “Yes, it was normal”, “Yes, but I do not know the result”, “No, I did not”.

Descriptive statistics are used for presentation of data; qualitative variables are presented as numbers (frequencies), while continuous variables are presented as mean (standard deviation). Kolmogorov-Smirnov and normality tests were used to check for the distribution of continuous variables. In order to estimate the relationship between two categorical variables chi-square test was used, while the chi-square trend test was used was used with regard to the relationship between an ordinal and a categorical variable. Also, for the investigation of the relationship between a continuous and a categorical variable the student's t-test or ANOVA was used. Logistic regression analyses were performed with the use of self-reported preventive health examinations of immigrants and taking medicines as the dependent variables. Finally, multivariate logistic regression with backward stepwise logistic regression was performed and the odds ratios, 95% confidence intervals and p values are presented. Data analysis was performed using IBM SPSS 21.0 and statistical significance level was set at 0.05.

## 3. Results

### 3.1. Demographic characteristics

The study population consisted of 1152 immigrants. In Table 1 participants' origin is presented in comparison to the data recorded by the 2011 census. In this study the majority (51.4%) originated from Albania. Three quarters of immigrants were living in Athens (46%) and Thessaloniki (27.3%), and the rest in major cities around the country (Ioannina (9.4%), Herakleion (6.9%), Volos (6.3%), Lamia (4.3%)).

**Table 1.** Origin of immigrants and comparison to the 2011 census.

Country of origin	Study sample (%)	Census 2011 (%)
Albania	592 (51.4)	480,824 (78.4)
Other eastern European countries	211 (18.3)	54,797 (9.0)
Asia	299 (26.0)	61,968 (10.2)
AfricaTotal	50 (4.3)1152 (100.0)	15,346 (2.4)612,935 (100.0)^a^

*Notes: ^a^ Sum presented refers to the specific nationalities.

The majority of immigrants (52.6%) were males with mean age 37.6 years, married (58.2%) and they had been living in Greece for an average of 10.9 years. Of those 63.5% were working, while 80.9% had legal documents for living and working in Greece. More than half of them(58.2%) had valid health and social security.

In Table 2 the demographic characteristics are shown according to the country/region of origin. Male sex was more frequent among Asians and Africans compared to Europeans, while females were the vast majority of those originating from former Soviet Union (SU) countries. Mean age and mean duration of living in Greece was higher for Europeans in comparison to Asians and Africans. Albanians and Africans had valid health and social security to a greater extent and Albanians were married at a larger percentage, while those originating from former SU countries had a higher level of education. Living conditions differed according to country of origin. In particular, Albanians and Africans were mostly living in families, Asians were mostly living with friends/colleagues/compatriots and former SU immigrants were mostly living with their employers.

**Table 2.** Demographic characteristics of the study sample (Ν = 1152).

Characteristic	Immigrants, n (%)					P-value
	Albanians	Asian	East Europeans	Africans	Total	
Sex						<0.001^a^
Male	290 (49.0)	234 (78.3)	47 (22.3)	35 (70.0)	606 (52.6)	
Female	302 (51.0)	65 (21.7)	164 (77.7)	15 (30.0)	546 (47.4)	
Age^b^	39.2 (11.3)	32.5 (10.7)	40.5 (12.3)	34.5 (8.6)	37.6 (11.6)	<0.001^c^
Duration of stay in Greece^b^	13.2 (5.7)	6.5 (6.5)	11.1 (6.3)	7.6 (5.7)	10.9 (6.7)	<0.001^c^
Valid health insurance						<0.001^a^
Yes	460 (77.7)	71 (23.7)	108 (51.2)	31 (62.0)	670 (58.2)	
No	132 (22.3)	228 (76.3)	103 (48.8)	19 (38,.0)	482 (41.8)	
Legal documents						<0.001^a^
Yes	552 (93.2)	189 (63.2)	148 (70.1)	43 (86.0)	932 (80.9)	
No	40 (6.8)	110 (36.8)	63 (29.9)	7 (14.0)	220 (19.1)	
Educational level						<0.001^d^
Up to 6 years of school	166 (28.0)	38 (12.7)	11 (5.2)	12 (24.0)	227 (19.7)	
Up to 9 years of school	163 (27.5)	77 (25.8)	33 (15.6)	15 (30.0)	288 (25.0)	
High school graduates	174 (29.4)	132 (44.1)	49 (23.2)	5 (10.0)	360 (31.3)	
Vocational education	41 (6.9)	13 (4.3)	40 (19.0)	6 (12.0)	100 (8.7)	
Higher education	48 (8.1)	39 (13.0)	78 (37.0)	12 (24.0)	177 (15.4)	
Number of individuals living in the same house^b^	3.0 (1.5)	5.1 (3.9)	2.4 (1.7)	2.8 (1.3)	3.4 (2.6)	<0.001^c^
Living with husband/partner						<0.001^a^
Yes	390 (65.9)	111 (37.1)	96 (45.5)	14 (28.0)	671 (58.2)	
No	202 (34.1)	188 (62.9)	115 (54.5)	36 (72.0)	481 (41.8)	
Living with children						<0.001^a^
Yes	340 (57.4)	88 (29.4)	63 (29.9)	10 (20.0)	501 (43.5)	
No	252 (42.6)	211 (70.6)	148 (70.1)	40 (80.0)	651 (56.5)	
Living with relatives						0.001^a^
Yes	107 (18.1)	41 (13.7)	41 (19.4)	19 (38.0)	208 (18.1)	
No	485 (81.9)	258 (86.3)	170 (80.6)	31 (62.0)	944 (81.9)	
Living with friends						<0.001^a^
Yes	33 (5.6)	138 (46.2)	12 (5.7)	18 (36.0)	201 (17.4)	
No	559 (94.4)	161 (53.8)	199 (94.3)	32 (64.0)	951 (82.6)	
Living with employer						<0.001^a^
Yes	1 (0.2)	10 (3.3)	27 (12.8)	0 (0.0)	38 (3.3)	
No	591 (99.8)	289 (96.7)	184 (87.2)	50 (100.0)	1114 (96.7)	
Living with colleagues						<0.001^a^
Yes	22 (3.7)	40 (13.4)	8 (3.8)	0 (0.0)	70 (6.1)	
No	570 (96.3)	259 (86.6)	203 (96.2)	50 (100.0)	1082 (93.9)	
Perception of access to health services in comparison with Greeks						<0.001^a^
Yes	191 (32.3)	142 (47.5)	61 (28.9)	26 (52.0)	420 (36.5)	
No	401 (67.7)	157 (52.5)	150 (71.1)	24 (48.0)	732 (63.5)	
Working at the time of the study						<0.001^a^
Yes	418 (70.6)	118 (39.5)	161 (76.3)	34 (68.0)	731 (63.5)	
No	174 (29.4)	181 (60.5)	50 (23.7)	16 (32.0)	421 (36.5)	
Permanent employment						<0.001^a^
Yes	254 (42.9)	63 (21.1)	92 (43.6)	15 (30.0)	424 (36.8)	
No	338 (57.1)	236 (78.9)	119 (56.4)	35 (70.0)	728 (63.2)	
Full-time employment						<0.001^a^
Yes	272 (45.9)	73 (24.4)	91 (43.1)	15 (30.0)	451 (39.1)	
No	320 (54.1)	226 (75.6)	120 (56.9)	35 (70.0)	701 (60.9)	
Support from family						<0.001^a^
Yes	493 (83.3)	121 (40.5)	125 (59.2)	32 (64.0)	771 (66.9)	
No	99 (16.7)	178 (59.5)	86 (40.8)	18 (36.0)	381 (33.1)	
Support from friends						<0.001^a^
Yes	512 (86.5)	146 (48.8)	141 (66.8)	40 (80.0)	839 (72.8)	
No	80 (13.5)	153 (51.2)	70 (33.2)	10 (20.0)	313 (27.2)	

*Notes: ^a^ chi-square test; ^b^ mean (standard deviation); ^c^ analysis of variance; ^d^ chi-square trend test.

### 3.2. Health status characteristics

In Table 3 data related to health characteristics are presented. Self-reported health was good/very good in 66.6% of immigrants, while 21.2% reported that their health was better/much better in comparison to the previous year. Smokers were 27.9% of the total study sample with average daily consumption of 19.1 cigarettes and mean duration of smoking of 13.7 years. Average exercise per week was 1.2 hours and alcohol consumption was 1.9 units per week. Albanians and Africans reported better health than East Europeans and Asians. Daily consumption of cigarettes was higher in Asians, but Albanians were smoking for a longer time. Alcohol consumption was higher in Albanians.

**Table 3.** Health status characteristics of immigrants according to country/region of origin.

Health characteristic	Immigrants, n (%)					P-value
	Albanians	Asians	East Europeans	Africans	Total	
Current self-reported health						<0.001^a^
Very poor	6 (1.0)	11 (3.7)	25 (11.8)	0 (0.0)	42 (3.6)	
Poor	12 (2.0)	18 (6.0)	11 (5.2)	1 (2.0)	42 (3.6)	
Average	102 (17.2)	133 (44.5)	51 (24.2)	14 (28.0)	300 (26.0)	
Good	280 (47.3)	70 (23.4)	76 (36.0)	25 (50.0)	451 (39.1)	
Very good	192 (32.4)	67 (22.4)	48 (22.7)	10 (20.0)	317 (27.5)	
Current self-reported health in comparison to previous year						0.1^a^
Much worse	6 (1.0)	9 (3.0)	5 (2.4)	0 (0.0)	20 (1.7)	
Worse	35 (5.9)	57 (19.1)	27 (12.8)	4 (8.0)	123 (10.7)	
Same	411 (69.4)	194 (64.9)	133 (63.0)	27 (54.0)	765 (66.4)	
Better	108 (18.2)	29 (9.7)	37 (17.5)	15 (30.0)	189 (16.4)	
Much better	32 (5.4)	10 (3.3)	9 (4.3)	4 (8.0)	55 (4.8)	
Smoking						0.1^a^
Current	165 (27.9)	98 (32.8)	47 (22.3)	11 (22.0)	321 (27.9)	
Former	40 (6.8)	10 (3.3)	13 (6.2)	2 (4.0)	65 (5.6)	
Never	387 (65.4)	191 (63.9)	151 (71.6)	37 (74.0)	766 (66.5)	
Daily cigarette consumption^b^	19.6 (10.0)	21.7 (8.9)	14.8 (7.4)	12.8 (5.0)	19.1 (9.5)	<0.001^c^
Years of smoking^b^	15.0 (9.2)	8.9 (6.2)	13.9 (9.3)	8.8 (6.2)	13.7 (8.8)	<0.001^c^
Hours of weekly exercise^b^	1.2 (3.2)	0.8 (1.8)	1.6 (3.3)	1.2 (1.8)	1.2 (2.9)	0.03^c^
Units of alcohol consumption per week^b^	2.7 (4.1)	0.8 (2.2)	1.3 (2.9)	1.7 (1.9)	1.9 (3.5)	<0.001^c^

*Notes: ^a^ chi-square trend test; ^b^ mean (standard deviation); ^c^ analysis of variance.

In Table 4 common health problems are reported. The most common health problem reported was hypertension (6.8%), followed by gastrointestinal problems (4.7%), cardiovascular disease (4%) and mental disease (4%). Medicines for chronic conditions were taken by 15.9% of immigrants, while 55.7% of them reported that they sometimes did not take their medication due to its cost. Asians had mental health problems and did not take their medicine due to its cost at a higher proportion.

**Table 4.** Self-reported health problems of immigrants according to country/region of origin.

Health problem	Immigrants, n (%)					P-value
	Albanians	Asians	East Europeans	Africans	Total	
Hypertension						0.1^a^
Yes	39 (6.6)	26 (8.7)	13 (6.2)	0 (0.0)	78 (6.8)	
No	553 (93.4)	273 (91.3)	198 (93.8)	50 (100.0)	1074 (93.2)	
Asthma						0.3^a^
Yes	19 (3.2)	15 (5.0)	7 (3.3)	0 (0.0)	41 (3.6)	
No	573 (96.8)	284 (95.0)	204 (96.7)	50 (100.0)	1111 (96.4)	
Diabetes						0.5^a^
Yes	18 (3.0)	9 (3.0)	9 (4.3)	0 (0.0)	36 (3.1)	
No	574 (97.0)	290 (97.0)	202 (95.7)	50 (100.0)	1116 (96.9)	
Cardiovascular disease						0.002^a^
Yes	11 (1.9)	18 (6.0)	15 (7.1)	2 (4.0)	46 (4.0)	
No	581 (98.1)	281 (94.0)	196 (92.9)	48 (96.0)	1106 (96.0)	
Gastrointestinal disease						0.01^a^
Yes	23 (3.9)	8 (2.7)	19 (9.0)	4 (8.0)	54 (4.7)	
No	569 (96.1)	291 (97.3)	191 (90.5)	46 (92.0)	1097 (95.2)	
Mental health disease						0.001^a^
Yes	6 (1.0)	34 (11.4)	6 (2.8)	0 (0.0)	46 (4.0)	
No	586 (99.0)	265 (88.6)	205 (97.2)	50 (100.0)	1106 (96.0)	
Medicines for chronic diseases						0.014^a^
Yes	83 (14.0)	43 (14.4)	49 (23.2)	8 (16.0)	183 (15.9)	
No	509 (86.0)	256 (85.6)	162 (76.8)	42 (84.0)	969 (84.1)	
Non taking a medicine due to cost						<0.001^a^
Yes	42 (50.6)	37 (86.0)	19 (38.8)	4 (50.0)	102 (55.7)	
No	41 (49.4)	6 (14.0)	30 (61.2)	4 (50.0)	81 (44.3)	

*Notes: ^a^ chi-square test.

In Table 5 the most common preventive tests undertaken by immigrants are reported. Two thirds (68.9%) of immigrants had been tested for blood count, 61.1% had their blood pressure measured, 56.8% had their cholesterol measured, 55.1% had their blood sugar measured. Albanians had been tested for blood count, blood pressure, blood glucose and cholesterol more frequently than all other groups of immigrants. African women were tested more frequently with pap smear and mammography. East European men had been tested more frequently for prostate.

**Table 5.** Self-reported preventive health examinations of immigrants according to country/region of origin.

Examination during the last 2 years	Immigrants, n (%)					P-value
	Albanians	Asians	East Europeans	Africans	Total	
Blood count						<0.001^a^
Measured and found normal	426 (72.0)	121 (40.5)	129 (61.1)	25 (50.0)	701 (60.9)	
Measured and found abnormal	33 (5.6)	7 (2.3)	22 (10.4)	3 (6.0)	65 (5.6)	
Not measured	125 (21.1)	155 (51.8)	58 (27.5)	20 (40.0)	358 (31.1)	
Measured but do not know the result	8 (1.4)	16 (5.4)	2 (0.9)	2 (4.0)	28 (2.4)	
Blood pressure						<0.001^a^
Measured and found normal	335 (56.6)	97 (32.4)	97 (46.0)	22 (44.0)	551 (47.8)	
Measured and found abnormal	60 (10.1)	40 (13.4)	31 (14.7)	4 (8.0)	135 (11.7)	
Not measured	188 (10.1)	155 (51.8)	83 (39.3)	22 (44.0)	448 (38.9)	
Measured but do not know the result	9 (1.5)	7 (2.3)	0 (0.0)	2 (4.0)	18 (1.6)	
Cholesterol						<0.001^a^
Measured and found normal	300 (50.7)	84 (28.1)	83 (39.3)	20 (40.0)	487 (42.3)	
Measured and found abnormal	77 (13.0)	45 (15.1)	27 (12.8)	0 (0.0)	149 (12.9)	
Not measured	206 (34.8)	164 (54.8)	100 (47.4)	28 (56.0)	498 (43.2)	
Measured but do not know the result	9 (1.5)	6 (2.0)	1 (0.5)	2 (4.0)	18 (1.6)	
Blood sugar						<0.001^a^
Measured and found normal	330 (55.7)	89 (29.8)	97 (46.0)	22 (44.0)	538 (46.7)	
Measured and found abnormal	28 (4.7)	40 (13.4)	13 (6.2)	0 (0.0)	81 (7.0)	
Not measured	227 (38.3)	165 (55.2)	99 (46.9)	26 (52.0)	517 (44.9)	
Measured but do not know the result	7 (1.2)	5 (1.7)	2 (0.9)	2 (4.0)	16 (1.4)	

*Notes: ^a^ chi-square test.

Logistic regression analyses with self-reported preventive health examinations of immigrants and taking medicines as the dependent variables are shown in Table 6. Immigrants without valid health insurance and worst self-reported health did not take more often their medicines due to cost. Also, decreased monthly family income was associated with higher probability of non-taking a medicine due to cost. Immigrants with legal documents and valid health insurance performed more often blood count measurement, blood pressure measurement, cholesterol measurement and blood sugar measurement. Also, increased monthly family income was associated with higher probability of blood count measurement. Very poor/poor/average current self-reported health and increased age were associated with higher probability of taking medicines for chronic diseases.

**Table 6.** Logistic regression analyses with self-reported preventive health examinations of immigrants and taking medicines as the dependent variables.

Dependent variable	Independent variables	Odds ratio	95% confidence interval	P-value
Non taking a medicine due to cost vs taking	Valid health insurance vs none health insurance	5.23	2.39 to 11.45	<0.001
	Monthly family income (0€ = reference category)^a^			<0.001
	>1000€	0.21	0.07 to 0.68	
	601–1000€	0.29	0.10 to 0.87	
	1–600€	0.15	0.04 to 0.54	
	Very poor/poor/average current self-reported health vs good/very good	2.67	1.29 to 5.44	<0.001
Blood count measurement vs no	Legal documents vs non legal	4.11	2.85 to 5.92	<0.001
	Valid health insurance vs none health insurance	3.11	2.28 to 4.23	<0.001
	Monthly family income (0€ = reference category)^a^			<0.001
	>1000€	3.40	2.06 to 5.60	
	601–1000€	1.66	1.24 to 2.74	
	1–600€	2.38	1.38 to 4.10	
Blood pressure measurement vs no	Legal documents vs non legal	4.09	2.82 to 5.93	<0.001
	Valid health insurance vs none health insurance	2.14	1.60 to 2.84	<0.001
Cholesterol measurement vs no	Legal documents vs non legal	4.65	3.12 to 6.91	<0.001
	Valid health insurance vs none health insurance	2.36	1.78 to 3.13	<0.001
Blood sugar measurement vs no	Legal documents vs non legal	4.37	2.95 to 6.47	<0.001
	Valid health insurance vs none health insurance	2.00	1.51 to 2.64	<0.001
Medicines for chronic diseases vs no	Very poor/poor/average current self-reported health vs good/very good	4.00	2.86 to 5.62	<0.001
	Age	1.05	1.03 to 1.06	<0.001

*Notes: ^a^ categories were created according to quartiles (25^th^ = 0€, 50^th^ = 600€ and 75^th^ = 1000€).

## 4. Discussion

Differences observed between the current study sample's ethnic origin and that of the 2011 census [1] is due to oversampling specific categories (e.g. Afghans), since this was necessary in order to make meaningful comparisons between immigrants from differed countries. However, this might have also been affected by the response rate, which was not particularly high, as some categories of immigrants were not willing to participate for various reasons, such as lack of interest or fear of losing their residence permit. Most of the participants were males, young adults, married, living in Greece for an average of ten years, in large cities, working and having legal documents. These findings are in accordance with other studies [13],[15],[16].

As it was expected immigrants were healthy, even though they presented some unhealthy habits such as smoking and inadequate exercising. These findings confirm the ones met in other studies performed with similar immigrant populations in Greece and elsewhere [13], [22].

Immigrants reported their health status as good/very good and the majority of them did not report health problems, especially those of Albanian origin. This finding is similar to that reported by Galanis et al. [16], Rapp et al. [23] and Malmusi & Ortiz-Barreda [6]. However, a systematic review has revealed that the immigrant population is exposed to lower socioeconomic status than natives, and despite a lower prevalence of chronic diseases, they seem to experience more mental health problems and worse self-reported health [6]. Similar results, regarding self-reported health and in particular mental health, were also reported in the study by Kuehne et al. [24] for undocumented migrants in Germany. These findings, despite referring to an even more disadvantaged group within the immigrant population, should provide us with further ideas for future explorative studies in order to formulate a clearer picture of the health problems migrants face in the host countries. However, even though health problems were not frequently reported, a considerable proportion of immigrants were using medication, even though they had difficulties in purchasing it due to cost, as it has been described by Kaitelidou et al. [17].

Regarding preventive tests, such as blood count, blood pressure, blood glucose and cholesterol, a significant proportion of immigrants did not adhere to them, although differences were found according to their ethnic origin. Again, immigrants from Albania reported undertaking preventive tests to a greater extent than the rest of the immigrant groups. This can be explained by the size of this group, contributing to the formation of an information and support network within their community. Similar findings have been reported by Bucaj [25] who studied Albanian immigrants in Greece and found that they did not adhere to general preventive tests and men even less frequently than women. Also, Simou et al. [12] found that immigrant women did not undergo preventive tests such as pap-smear test and mammogram. Similar findings have been reported in an Austrian study, in which immigrants of both sexes from countries outside the EU had the lowest adherence in general preventive tests [26]. These findings however, need further study in order to find out if undergoing preventive tests is influenced by other factors such as the immigrants' perception of preventive health care according to their ethnic culture. At the time of this study information regarding access to preventive health care was not widely available in immigrants' national languages, an issue that may have also affected immigrants' use of such services.

In our study, factors that seem to influence undergoing preventive tests were valid legal documents and health insurance. This finding is supported by the literature [24],[25], although in some studies other factors also seem to contribute favorably, such as education and income [26]. As far as medication is concerned, the same factors apply, with the addition of age, considering the chronicity of the health problem. This finding is in accordance with other studies [6],[17],[27],[28] as it is well documented in literature that health coverage is one of the most significant factors affecting access to health care services for vulnerable populations including migrants [29]. It should be noted however, that at the time of this study, employment was linked with the right to access health care and therefore access to care for the uninsured population including migrants, was quite limited.

The evidence provided from this study supports the findings of other studies performed in Greece and elsewhere, meaning that the major problem related to health needs of immigrants is access to health care services with regard to either health promotion and prevention or managing chronic health problems. In addition to that, immigrants' age also affects the use of health services; this is of great importance because as they get older their health problems constitute a greater burden if access is not adequate. Ethnic origin also seems to influence the use of preventive health services, a finding that could guide planning of such services for different groups of immigrants, including the availability of information in immigrants' own language.

It is important to note that this study provides some evidence on the health care needs of immigrants living in Greece; further research is needed in order to investigate the needs of immigrants and refugees with different ethnic characteristics that continue flowing in the country. Such information will be useful not only for Greece but so as to also inform European countries' health systems that provide support to migrant populations. Robust epidemiological data are of prime importance for the support of effective disease surveillance and reporting systems, early diagnosis and treatment of clinical syndromes, prevention of communicable diseases and effective chronic disease management. Arrival, transit and destination countries should possess all the above mentioned information in order to better prepare their health systems to address the needs of the immigrant populations. So far, there is an evident lack of data regarding health status, health needs and the different attitudes towards health and health care by migrant populations. Therefore, the systematic measurement of health status is an imperative need for Greece, as it is in other European countries as well and requires a quite complex and multidimensional procedure which includes apart from morbidity indicators, non-medical determinants of health which result in lifestyle diseases, qualitative measures of quality of life as well as cultural influences on health behaviors, which may have a significant impact on health promotion campaigns and a differential choice of health-care strategies.

Our study had several limitations. A cross-sectional study provides only a snapshot of the current situation since changes over time are probable. Snowball sampling limits generalization of results, as it increases the possibility of systematic bias, although it gives a general idea of the extent of the problem in the population under study. Also, data were self-reported by means of a questionnaire and there may be an information bias.

## 5. Conclusion

In conclusion, immigrants living in Greece for a considerable time report good health and their self-reported perceived health is also good. This information should be taken into account when health promotion and prevention programs are designed for this population. In addition to that, further research is needed in order to ensure that such programs are beneficial not only for the populations included in this study, but also for immigrants and refugees that continue flowing in Greece.
